# Simple Sugar Intake and Hepatocellular Carcinoma: Epidemiological and Mechanistic Insight

**DOI:** 10.3390/nu6125933

**Published:** 2014-12-22

**Authors:** Juan Carlos Laguna, Marta Alegret, Núria Roglans

**Affiliations:** 1 Unit of Pharmacology, Department of Pharmacology and Therapeutic Chemistry, School of Pharmacy, University of Barcelona, Avda. Joan XXIII s.n., 08028 Barcelona, Spain; E-Mails: alegret@ub.edu (M.A.); roglans@ub.edu (N.R.); 2 Institute of Biomedicine (IBUB), University of Barcelona, Barcelona 08028, Spain; 3 Spanish Biomedical Research Centre in Physiopathology of Obesity and Nutrition, Institute of Health Carlos III, Spanish Government, Diet and Atherosclerosis Node, Barcelona 08036, Spain

**Keywords:** fructose, high-fructose corn syrup, hepatocellular carcinoma, non-alcoholic fatty liver disease (NAFLD), obesity, insulin resistance

## Abstract

Sugar intake has dramatically increased during the last few decades. Specifically, there has been a clear trend towards higher consumption of fructose and high fructose corn syrup, which are the most common added sugars in processed food, soft drinks and other sweetened beverages. Although still controversial, this rising trend in simple sugar consumption has been positively associated with weight gain and obesity, insulin resistance and type 2 diabetes mellitus and non-alcoholic fatty liver disease. Interestingly, all of these metabolic alterations have also been related to the development of hepatocellular carcinoma. The purpose of this review is to discuss the evidence coming from epidemiological studies and data from animal models relating the consumption of simple sugars, and specifically fructose, with an increased risk of hepatocellular carcinoma and to gain insight into the putative molecular mechanisms involved.

## 1. Introduction

Non-communicable diseases continue to pose an enormous burden on humanity, having recently surpassed infectious diseases as the primary concern for human health [1]. Cardiovascular events derived from the development of atherosclerosis associated with chronic metabolic diseases, such as obesity, type 2 diabetes mellitus (T2DM) and metabolic syndrome, are increasing all over the world. Besides genetic predisposition, two life-style characteristics deeply rooted in affluent Western societies are fuelling this vicious trend: a lack of physical activity and the consumption of hypercaloric diets, tipping the energy balance of individuals to an excess of ingested, and not used, energy. This global change in dietary habits is related to the introduction by the food industry of fructose as a sweetener, either as an integrant of the sucrose molecule (in European countries) or as a component of the so-called “high-fructose corn syrups” (in the United States). The relationship between excessive fructose consumption and obesity has been established in humans. Obesity clusters with other metabolic dysfunctions (dyslipidemia, hypertension, non-alcoholic fatty liver disease (NAFLD), insulin resistance and T2DM) in metabolic syndrome, and fructose consumption has also been linked with these clinical entities.

NAFLD is the hepatic manifestation of metabolic syndrome, and its appearance and development is directly related to a deficit in insulin signaling, both in the liver and the whole organism. NAFLD is a spectrum of disorders ranging from simple steatosis to non-alcoholic steatohepatitis (NASH) and cirrhosis. NASH appears to portend an increased risk of hepatocellular carcinoma (HCC), the sixth most common cancer worldwide, the major histological subtype of primary liver malignancies and the third most frequent cause of death related to cancer. Further, sustained hyperinsulinemia and T2DM are associated with an increased prevalence of several types of cancer, including HCC. Thus, high simple sugar consumption is a known risk factor for obesity, NAFLD, insulin resistance and T2DM, and these pathologies are also recognized risk factors for the development of HCC. Here, we attempt to comprehensively review epidemiological data relating simple sugar intake and risk of HCC, with a special focus on describing what we know about possible molecular mechanisms that could explain this association.

## 2. Insulin Resistance, Hyperinsulinemia and HCC

Several cohort and case-control studies and meta-analyses have shown an association between diabetes, metabolic syndrome and HCC among human populations of different geographical origin [2,3,4,5]. Moreover, poor glycemic control seems to be a significant prognostic factor of HCC after curative therapy in diabetic patients [6]. Although not all published data are consistent with the existence of this association (see data on a Swedish population reported by Miao Jonas son, *et al.*) [7], the Emerging Risk Factors Collaboration [8] has recently demonstrated an increased hazards ratio for cancer-related death (1.80, 95% confidence interval 1.71 to 1.90) among persons with diabetes compared with persons without diabetes, after adjusting for age, sex, smoking status and body-mass index, having analyzed data on 123,205 deaths among 820,900 people. Interestingly, the highest hazards ratio for individual types of cancer corresponded to that of HCC (2.16, CI 1.62 to 2.88).

Recent data point to an association between the increased prevalence of several types of cancer in type 2 diabetic patients and sustained hyperinsulinemia associated with an increased rate of insulin production, reflected by plasma C-peptide levels [9,10]. The insulin receptor is a member of the insulin growth factor receptor (IGFR) family of membrane tyrosine kinase receptors, which bind not only insulin, but also insulin-like growth factors (IGF) and are homologous to the tyrosine kinase class of oncogenes [11]. These receptors are present not only in cells from classical insulin-sensitive tissues, such as liver, skeletal or adipose tissue, but also in many normal and transformed epithelial cells. Assuming that transformed cells remain insulin sensitive in a situation of classical insulin resistance (diabetes, metabolic syndrome) and given that, as a rule, the expression or intrinsic activity of IGFR is not changed in many types of cancers, their growth will be stimulated by the ensuing hyperinsulinemia [12]. Furthermore, hyperinsulinemia also increases the concentration of free, active IGF-1 in blood, thus further potentiating the stimulation of IGFR [13].

Downstream in the signaling pathway of the insulin receptor and many other tyrosine kinase receptors is the Ras-Raf-MEK-MAPK mitogenic pathway and the phosphatidylinositol-4,5-bisphosphate 3-kinase (PI3K)—V-akt murine thymoma viral oncogene (Akt)–mammalian target of rapamycin (mTOR) cascade, which are involved in the production of many proliferative and survival signals for precancerous and cancerous cells [12,14]. mTOR is a key factor in the energy-sensing system of the cells; when energy is available, mTOR activation upregulates several anabolic, energy-consuming pathways (lipogenesis, protein synthesis, *etc.*), controlling mRNA translation and ribosome biogenesis [15]. Thus, in hyperinsulinemic individuals, the continuous activity of insulin in non-resistant tissues promotes the activation of the mTOR system, thereby favoring cellular proliferation and tumor development [16]. Metformin, an insulin sensitizer drug that is associated in several epidemiological studies with a reduction in cancer incidence, is able to reduce the degree of mTOR activation in many cancer cell lines [17].

## 3. Obesity and HCC

The prevalence of obesity, defined as having a body mass index (BMI) ≥30 kg/m^2^, has been increasing worldwide in recent decades and has become a huge public health problem. This is due to the close association between obesity and conditions, such as insulin resistance, diabetes and hypertension, which cluster in metabolic syndrome, and also due to the fact that obesity increases the risk of many types of cancer, including liver cancer [18]. The rise in prevalence of obesity has coincided with an increase in the incidence of hepatic cancer in the United States since the mid-1980s, and several epidemiological studies have demonstrated the link between obesity and the risk of HCC [5,19,20]. Two excellent reviews on the epidemiological, experimental and clinical evidence linking obesity and HCC have been published [18,21]. One of the larger prospective studies performed, including more than 900,000 individuals from the USA, showed that obese men with a BMI ≥35 kg/m^2^ have a relative risk of HCC of 4.5 compared with those with a normal BMI [22]. A meta-analysis of 11 cohort studies showed that compared to persons of normal weight, overweight or obese individuals had a 17 and 89% increased risk of hepatic cancer, respectively [23]. The study also showed that the risk was higher for men than for women. A very recent and large-scale cohort study involving 5.2 million individuals from the U.K. Clinical Practice Research Datalink showed that, after adjusting for potential confounders, there was an overall positive association between BMI and liver cancer. With the assumption that the association was causal, it was estimated that 15.6% of new cases of liver cancer per year were attributable to being overweight and obesity [24]. On the other hand, it has been shown that obesity synergizes with other etiologies, such as excessive alcohol consumption, to increase the risk of incident HCC [25].

Mechanistically, hepatocarcinogenesis in obesity-associated liver disease may be related to the expansion of adipose tissue, its remodeling and the resulting low-grade chronic inflammation, characterized by an altered pattern of adipokine secretion, with increased levels of leptin and inflammatory cytokines and decreased adiponectin. Moreover, the link between obesity and HCC may be accomplished through NAFLD, as it has been reported that 70%–80% of obese individuals have a fatty liver [21].

## 4. NAFLD and HCC

NAFLD is the most common hepatic manifestation of obesity and metabolic syndrome, affecting at least 20%–30% of adults [26,27]. The initial and most benign manifestation of NAFLD is the simple accumulation of triglycerides in the hepatocyte (hepatic steatosis). However, in a subset of patients, this condition can progress to a more serious disease, characterized by hepatic necroinflammation (NASH). Although the results are not conclusive, due to the lack of noninvasive tests that can differentiate between the two conditions, it has been suggested that 10%–25% of patients with hepatic steatosis develop NASH [27,28]. Progression to NASH was formerly explained by the “two-hit” theory [29], which proposed that hepatic fat accumulation acts as a first hit, sensitizing the liver to inflammatory mediators and oxidative stress (second hit insults). This initial view has recently been challenged by the so-called “multi-hit” or “parallel-hit” theory, where the first hit is insulin resistance, which causes hyperinsulinemia, increased hepatic *de novo* lipogenesis and impaired inhibition of adipose tissue lipolysis, which results in an increased flux of free fatty acids to the liver and hepatic fat infiltration. This fatty liver becomes extremely vulnerable to a complex series of second hits, including the formation of oxidized lipid by-products, dysregulated hepatocyte apoptosis and multiple pro-inflammatory and pro-fibrotic mediators, resulting in progression to NASH [30]. NASH can occur without collagen deposition, but in 15%–25% of patients, lesions may progress to hepatic fibrosis and cirrhosis [28,30].

Several epidemiological studies have shown that NAFLD increases the risk of HCC [28]. According to these studies, the percentage of cases of HCC caused by NAFLD ranges between 4% and 22% in Western countries, but it should be noted that most of them were performed in relatively small patient populations. The biggest study to date is a retrospective study from Japan, which included 14,530 patients with HCC [31]. The results of this study showed that the prevalence of NAFLD-related HCC may be lower in Asian than in Western countries, probably due to the higher prevalence of HCC related to hepatitis C virus in Asia.

The evolution of NASH to cirrhosis is one of the factors that may potentially explain the link between NAFLD and HCC, as the risk is higher when only patients with NAFLD-related cirrhosis are considered than when patients with all stages of NAFLD are studied [21]. However, liver cancer may also arise in patients with NAFLD in the absence of cirrhosis [21,28], suggesting that other mechanisms could play a significant role. It should be taken into account that metabolic alterations strongly linked to NAFLD (obesity and type 2 diabetes) are also risk factors for HCC. Moreover, diabetes not only increases the risk of HCC as an independent factor, but also potentiates the obesity-associated risk of HCC [32].

Several mediators and pathways have been proposed to link NAFLD and NASH to HCC. The most obvious are lipids themselves, as their ectopic deposition in tissues, such as the liver, causes cellular dysfunction, a process termed lipotoxicity, and this dysfunction may contribute to the development of HCC [33]. The mechanisms include, for example, the pro-oncogenic effects of fatty acids, the oxidative damage caused by lipid oxidation products and free radicals and the release of pro-inflammatory mediators.

## 5. Sugar Intake and Cancer Incidence

To date, only two reviews have examined the epidemiological and experimental data supporting a relationship between increased dietary fructose and cancer risk [34,35]. Epidemiological evidence linking dietary sugar, and, specifically, fructose consumption, with cancer is presented in Table 1. Two case-control studies with a retrospective evaluation of diet concluded that a high dietary glycemic load is associated with an increased risk for HCC, especially in patients with chronic viral hepatitis, and that a reduction of glycemic load in those patients could reduce the risk or delay the development of HCC [36,37]. More significant are the results arising from large prospective studies, such as those based on the NIH-AARP Diet and Health Study cohort. Among them, Jiao *et al.* (2009) [38] observed that free fructose consumption significantly increased the risk of pancreatic cancer, an observation that was confirmed later by a recent meta-analysis [39]. In the same NIH-AARP cohort, Tasevska *et al.* concluded that added fructose in the diet was associated with an increased risk of small intestine cancer [40]. Surprisingly, results from this study suggested that high added fructose intake in women was associated with a decrease in the risk of liver cancer, an effect that was attributed to an unidentified confounder [40]. The most recent and compelling evidence relating sugar consumption to cancer risk has come from the European Prospective Investigation into Cancer and Nutrition cohort, which included more than 477,000 individuals [41]. In this study, a positive association was found between increased total sugar intake and risk of HCC (hazards ratio adjusted for multiple variables 1.45 per 50 g/day). However, in a recently published study based on the NIH-AARP cohort, although fructose consumption was associated with an increase in all-cause mortality and cardiovascular disease mortality, the intake of individual sugars or added sugars was not associated with cancer mortality [42].

## 6. Simple Sugars, Insulin Resistance, Obesity, NAFLD and HCC: What Mechanisms Are Involved?

As discussed above, insulin resistance, obesity and NAFLD may lead to the establishment of HCC. In this section, we will focus on the mechanisms by which simple sugars, mainly fructose, induce these metabolic alterations and how they are linked to HCC development.

**Table 1:** Overview of human studies focused on cancer development and simple sugar consumption.

Authors	Type of Cancer	Study Characteristics	Sugar	Main Results
Aune *et al.* [39]	Pancreatic	Meta-analysis of ten cohort studies	GI, GL, TCH, S, F	Positive association with F intake (RR 1.22 per 25 g/day)
Bao *et al.* [43]	Pancreatic	Prospective study in 487,922 participants (NIH-AARP Diet and Health Study), 7.2 years follow-up	S, SSB	No association
Boyle *et al.* [44]	Cancer in general	Meta-analysis and review	SSB	No association
Chan *et al.* [45]	Pancreatic	Population based case (532) control (1701) study	SSB, F, L, SU	Positive association only with L (OR 2.0 comparing extreme quartiles)
Drake *et al.* [46]	Prostate	Prospective study in 8128 men	TCH, SSB	Positive association with SSB (HR 1.38 between highest and lowest consumption)
Fedirko *et al.* [41]	Liver and biliary tract	Prospective study in 477,206 participants	GI, GL, TCH	Positive association for TCH and HCC (HR: 1.45 per 50 g/day)
Gallus *et al.* [47]	Pancreatic	Combined data of a case (326) control (659) Italian study and other studies appearing before June, 2010	SSB	No association
Genkinger *et al.* [48]	Pancreatic	Pooled analysis of 14 prospective cohort studies (853,894 participants)	SSB	Positive small association (RR 1.06) with SSB when modeled as a continuous variable
Jackson *et al.* [49]	Prostate	Case (243) control (273) study	RCH (including SSB)	Positive association (OR 2.02) between highest and lowest tertiles of consumption
Jiao *et al.* [38]	Pancreatic	Prospective study in 482,362 participants (NIH-AARP Diet and Health Study), 11 years follow-up	GI, GL, TCH, ST, F, G	Positive association with F (RR 1.35) and G (RR 1.29) between highest and lowest quintiles of consumption
Lagiou *et al.* [36]	HCC	Case (333) control (360) study	GL	Positive association (OR 1.92) between highest and lowest quintiles in patients with chronic HBV and/or HCV infection
Larsson *et al.* [50]	Pancreatic	Prospective study in 77,797 participants, 8 years follow-up	S, SSB	Positive association, highest with SSB (HR 1.93 between highest and lowest consumption)
Mueller *et al.* [51]	Pancreatic	Prospective study in 60,524 participants (Singapore Chinese Health Study), 14 years follow-up	SSB	Positive association (HR 2.0 between highest and lowest consumption)
Rossi *et al.* [37]	HCC	Case (185) control (412) study	GL	Positive association (OR 3.02) between highest and lowest quintiles
Schernhammer *et al.* [52]	Pancreatic	Prospective study in 138,158 participants, 20 years follow-up	SSB	Positive association in women with an underlying degree of insulin resistance
Schernhammer *et al.* [53]	Lymphoma and leukemia	Prospective study in 138,158 participants, 22 years follow-up	SSB	Positive association (RR 1.66) with higher consumption of SSB in men
Tasevska *et al.* [40]	Cancer in general	Prospective study in 435,674 participants (NIH-AARP Diet and Health Study), 7.2 years follow-up	S, F, SU	Positive association of F with small intestine cancer (HR 2.20 between highest and lowest quintiles of consumption); Negative association of all sugars with ovarian cancer
Tasevska *et al.* [42]	Cancer-related mortality	Prospective study in 353,751 participants (NIH-AARP Diet and Health Study), 13 years follow-up	S,F,SU	No association between intake of individual or added sugars with cancer mortality
Theodoratou *et al.* [54]	Colon	Case (2062) control (2778) study	SSB	Positive association
Witte *et al.* [55]	Breast	Familial matched case (140) control study	TCH, SSB	Positive association with SSB
Zhang *et al.* [56]	Colon	Pooled data from 13 cohort studies (731,441 participants), 6–20 years follow-up	SSB	No association

F, fructose; G, glucose; GI, Glycemic Index; GL, glycemic load; HBV, hepatitis B virus; HCC, hepatocellular carcinoma; HCV, hepatitis C virus; HR, hazards ratio; L, lactose; OR, odds ratio; RCH, refined carbohydrates; RR, risks ratio; S, sugar; SSB, sugar-sweetened beverages; ST, starch; SU, sucrose; TCH, total carbohydrate.

### 6.1. Fructose and NAFLD

Regarding NAFLD, studies in rodents [57,58,59,60,61,62] and humans [63,64,65] have demonstrated that high-fructose diets cause hepatic steatosis. Fructose-induced lipid deposition in the liver has been attributed to the ability of this carbohydrate to increase hepatic *de novo* lipogenesis. Fructose is one of the carbohydrates with major lipogenic potential. This is because, unlike glucose, fructose hepatic metabolism bypasses the control step to enter the glycolytic pathway and therefore serves as an unregulated source of both triglyceride precursors, acetyl-CoA and glycerol 3-phosphate [66]. However, increased lipogenesis does not suffice to cause hypertriglyceridemia and liver steatosis, at least in rats. Our research group has demonstrated that in rats supplemented with 10% glucose or fructose, both sugars increased the hepatic synthesis of fatty acids, but only fructose increased the plasma and liver triglyceride content [57]. The difference between the two carbohydrates was that fructose, but not glucose, decreased hepatic fatty acid oxidation in addition to increasing lipogenesis. The reduction in fatty acid oxidation in male or female rats treated for two weeks with 10% liquid fructose was due to the inhibition of the expression and activity of peroxisome proliferator-activated receptor α (PPARα), a transcription factor that controls hepatic fatty acid catabolism [57,62]. However, fructose supplementation at the same concentration for longer periods (eight weeks) increases fatty acid synthesis and causes fatty liver, but does not reduce PPARα expression in the liver [67]. In this situation, hepatic fat accumulation seems to be related to a decrease in autophagic activity. Autophagy is a homeostatic mechanism through which the cytoplasmic components of the cell, including lipids, can be degraded to obtain energy to meet the bioenergetic demands of the cell under nutrient-poor conditions. Reduced autophagy has been linked to triglyceride accumulation in the hepatocytes [68], and on the other hand, intracellular lipid accumulation has been shown to inhibit autophagy, resulting in obesity and fatty liver [68,69]. Therefore, the inhibition of autophagy in the liver of rats supplemented with 10% liquid fructose for eight weeks may be responsible, at least in part, for the observed hepatic steatosis, and conversely, the accumulation of lipids in the liver may further contribute to the reduction of autophagy [67].

In addition to the increase in lipid synthesis, fructose contributes to hepatic lipid buildup through effects on plasma lipoproteins, specifically by increasing the production and reducing the clearance of very low density lipoproteins (VLDL). Thus, it has been demonstrated in both animals and humans that the metabolism of large amounts of fructose increases triglyceride synthesis and facilitates its esterification and release from the liver into the plasma in the form of VLDL [70]. In addition, it has been demonstrated in fructose-fed rats that there is also an impairment of VLDL catabolism [71], through reduced hydrolysis in peripheral tissues and impaired hepatic clearance of these lipoproteins [72].

According to the “two-hit” and “parallel-hit” theories, fructose, by inducing hepatic steatosis, would sensitize the liver to a series of secondary hits (lipid peroxidation products, inflammatory and pro-fibrotic mediators) that would result in the progression to NASH and HCC. Fructose consumption may also activate secondary hits by directly inducing a pro-inflammatory state. However, the effects of fructose on inflammatory mediators seem to depend on the animal species studied. A research group in Germany has carried out extensive work on mice supplemented with 30% liquid fructose [73,74,75,76,77,78], and their results support the notion that this sugar, by altering intestinal barrier permeability, causes a higher flux of endotoxin to the liver associated with steatosis, induced IκBα phosphorylation (indicative of NFκB activation) and increased TNFα levels in the liver. However, our preliminary results in female rats supplemented with 10% fructose indicate that in this model, hepatic steatosis is not associated with an increase in plasma endotoxin levels or the expression of inflammatory markers, such as TNFα, toll-like receptor-4 (TLR4) and monocyte chemoattractant protein-1 (MCP-1), in the liver.

In a recent study performed in 341 adults enrolled in the NASH Clinical Research Network, daily fructose ingestion was found to be associated with increased liver fibrosis in patients with NAFLD [79]. The authors suggested that the effects of fructose in the progression of liver damage could be in part explained through the exacerbation of abnormalities in hepatic fructose metabolism. In humans, rapid fructose phosphorylation to fructose 1-phosphate in the liver leads to an abrupt reduction in ATP and to an increase in AMP levels [80]. AMP can be recycled to ATP or metabolized to uric acid through xanthine dehydrogenase. In NASH patients, AMP is preferentially converted to uric acid, which exerts detrimental pro-inflammatory and pro-oxidative effects. Under these conditions, hepatocytes may be more vulnerable to ATP depletion, and the resulting impairment in energy homeostasis could also relate to the observed increase in hepatic inflammation.

The importance of pro-inflammatory mediators has been highlighted by authors, such as Wang *et al.* [81], who examined whether NASH induced by a high fat diet promoted hepatocarcinogenesis in rats injected with the hepatic carcinogen, diethylnitrosamine (DEN). Their results showed that compared with rats fed a control diet, those fed the high-fat diet developed a higher incidence and multiplicity of precancerous markers, together with a higher inflammatory response characterized by abundant infiltration of inflammatory cells and higher expression of TNF-α and NFκB [81]. TNFα acts as an activator of pro-oncogenic pathways, including mTOR and extracellular signal-regulated kinases [33]. On the other hand, activation of NFκB may promote cancer through upregulation of cyclin D1, an essential regulator of the cell cycle at the G1-S phase, or through its protective role against apoptosis, which would favor the survival of hepatocytes with genetic mutations [81]. According to this mechanism, Wunderlich *et al.* [82] showed in mice that the deficiency of the NFκB essential modulator, which activates NFκB, synergizes with a high-fat diet in the development of liver steatosis, causing elevated apoptosis and liver tumorigenesis.

To gain insight into the mechanisms by which fructose-induced fatty liver leads to the establishment of HCC, Kumamoto *et al.* [83] used rats fed either a control diet, a high-fat diet or a fructose-enriched diet (containing 66% of fructose in solid form) and concomitantly exposed to the hepatocarcinogen, DEN, in drinking water. They observed that although hepatic steatosis was more severe in the high-fat group than in the high-fructose group, the incidence of precancerous lesions of HCC was higher in the fructose group than in the other groups. On the contrary, fasting blood glucose and insulin levels were significantly higher in the high-fructose group. These results suggested that the mechanism by which fructose may contribute to HCC is related to insulin resistance rather than to hepatic steatosis.

Most studies about the effect of dietary components on NASH and HCC are based on the use of carcinogens, such as DEN [81,83,84] or N-nitrosomorpholine [85]. A recent study evaluated whether an adipogenic diet alone was sufficient to induce HCC in wild-type mice [86], avoiding the use of carcinogens, to simulate a more realistic situation. To this end, they used the American Lifestyle-Induced Obesity Syndrome (ALIOS) model, based on feeding mice with a diet containing trans-fatty acids (30% of the fat in the form of partially hydrogenated vegetable oil), adding 4.5% high fructose corn syrup to drinking water and combining it with a sedentary lifestyle. The results showed that the ALIOS mice developed NASH after six months and showed signs of advanced hepatic pathology (including fibrosis) at 12 months. At this age, hepatocellular neoplasms with features of HCC had developed in six of 10 ALIOS mice. Despite the interesting outcome of this study, indicating that chronic exposure to an unhealthy lifestyle is sufficient to cause HCC, the combined use of trans-fatty acids and HFCS precluded the discrimination of the specific effects of fructose on the initiation, promotion or development of HCC.

### 6.2. Fructose and Insulin Resistance

Fructose supplementation induces hyperinsulinemia and insulin resistance in animal models, shown by the inhibition of different steps in the insulin signaling pathway: reduced tyrosine phosphorylation of the insulin receptor and of insulin receptor substrates (IRS), reduced association of IRS with PI3K and/or inhibition of PI3K activity and Akt phosphorylation [80]. The response to fructose consumption may depend on gender, as we observed that after 14 days of supplementation with 10% fructose, only female rats developed clear signs of glucose intolerance and reduced expression of IRS-2 [59]. In a recent paper, we showed that this reduction in IRS-2 could be related to an increase in the phosphorylated, active form of mTOR, which has been shown to cause IRS-2 degradation [87]. mTOR exists in two distinct multi-protein complexes, of which mTORC1 is the primary nutrient-sensing complex. Insulin and other growth factors, such as IGF-1, signal through the PI3K/Akt/mTORC1 pathway, as activated Akt phosphorylates and inactivates the tuberous sclerosis protein 2 (TSC2), leading to mTORC1 activation. However, continued mTORC1 activation may favor insulin resistance by feedback inhibitory phosphorylation of IRS1 by mTORC1 or its downstream target, S6K [88,89,90]. Thus, either through IRS degradation or inhibitory phosphorylation, activation of mTOR by fructose may induce insulin resistance.

As mentioned above, mTOR activation has also been associated with cancer development [14,16]. Specifically in human HCC, mTOR activation correlates with tumor size, an aggressive phenotype and poor prognostic features [91,92]. The molecular mechanisms by which mTOR activation may favor HCC development include the induction/activation of angiogenesis-related proteins, such as hypoxia-inducible factor 1α and vascular endothelial growth factor, increased cellular proliferation through 4E-BP1, preferential translation of tumor-related genes, upregulation of lipogenesis and of glycolysis and suppression of autophagy [93].

When considering lipogenesis, it must be taken into account that while normal tissues usually rely on external sources to obtain lipids for structural synthesis, cancer cells preferentially use *de novo* fatty acid synthesis. In a context of elevated cell proliferation, increased lipogenesis is necessary to maintain a sufficient supply of lipids and lipid precursors to support membrane formation and post-translational modification of proteins. Induction of lipogenesis may be achieved in HCC through activation of the Akt/mTOR axis, which causes an increase in transcription and processing of sterol response element binding protein (SREBP)-1 and the subsequent increase in the expression of several lipogenic genes, including acetyl-CoA carboxylase (ACC), fatty acid synthase (FAS) and stearoyl-CoA desaturase (SCD) 1 [94]. Interestingly, we have found that 10% fructose supplementation to female rats increased the hepatic expression of FAS, ACC and other lipogenic enzymes, not through SREBP-1 activation, but through the induction of carbohydrate response element binding protein (ChREBP), a transcription factor responsible for inducing liver lipogenesis after carbohydrate ingestion [62]. It remains to be established whether activation of this pathway is also involved in HCC development.

Defective autophagy has also been related to cancer development due to its role as a tumor suppressor in early phases of tumorigenesis. Thus, it has been shown that expression of beclin-1, an important gene in the autophagic machinery, reduces tumorigenic capacity through induction of autophagy [95]. It has been demonstrated that beclin-1 knockout mice had an increased incidence of several cancers, including HCC [96], and a recent study showed lower expression of beclin-1 in human HCC tissues compared with the adjacent non-tumor regions [97]. These authors concluded that autophagy defects may be associated with the tumor progression and poor prognosis of HCC. As mentioned earlier, we have recently observed that supplementation with 10% fructose to female rats for eight weeks inhibited hepatic autophagy, shown by the reduction in the autophagic marker, LC3-II, through activation of mTORC1 [67]. This opens the possibility that fructose supplementation may also promote HCC development by mTORC1-driven suppression of autophagy.

### 6.3. Fructose and Obesity

In previous reviews, we gathered information from both animal and human studies on the effects of sugar-sweetened beverage consumption on weight gain and obesity [80,98]. Most studies based on food frequency questionnaires as an assessment method, as well as intervention studies have found a positive association between the consumption of these beverages and weight gain. Although we did not observe any increase in weight gain in rats supplemented with liquid fructose for 14 days in our previous studies [58,59,99], our recent results in female rats receiving 10% liquid fructose for eight weeks showed not only increased body weight, but also a significant increase in visceral adipose tissue mass [67]. Alzamendi *et al.* also found an increase in adipose tissue mass in male Wistar rats treated with 10% liquid fructose for three weeks [100]. This effect was accompanied by a decrease in the number of cells per gram of adipose tissue, while cell diameter and volume were significantly increased by fructose. Similarly, Creszenzo *et al.* observed differences in adipocyte number and morphology in male rats fed 30% fructose in solid form for eight weeks [101]. Thus, the number of intra-abdominal adipocytes was reduced due to an increase in their mean diameter, while the opposite (increased number, but decreased adipocyte size) was found for the subcutaneous abdominal depot.

As mentioned above, expanded adipose tissue releases pro-inflammatory cytokines, such as tumor TNF-α, NFκB and IL-6, which may play an important role in obesity-associated hepatocarcinogenesis [33,102]. To gain insight into these mechanisms, Park *et al.* [84] investigated whether dietary or genetic obesity was a direct promoter of HCC development in mice injected with a low dose of the hepatic carcinogen, DEN. Their results support the notion that obesity promotes hepatic tumors through induction of TNFα and IL-6, even under conditions in which DEN administration does not induce HCC on its own [84]. A role for IL-6 in hepatic cancer was proposed earlier by Naugler *et al.* [103], who observed that male rats developed more HCC than female rats after DEN administration, proposed that this gender difference (which was also observed in human studies showing that males have a higher HCC incidence than females [102], could be attributed to lower IL-6 production in females. IL-6 is pro-inflammatory, promotes steatohepatitis and activates signal transducer and activator of transcription 3 (STAT3) in hepatocytes, and all three mechanisms may be related to cancer induction. However, neither obesity nor activation of IL-6/STAT3 cause cancer on their own, but increase the possibility of progression to cancer of hepatocytes that have earlier acquired an oncogenic mutation [84].

Based on these mechanisms, one could speculate that fructose consumption influences HCC development through adipose tissue mass expansion and increased release of pro-inflammatory mediators. However, Velickovic *et al.* [104] observed that TNFα expression was not increased in the adipose tissue of rats fed a diet containing a 10% fructose solution in drinking water for nine weeks. The authors attributed this lack of effect to the increased level of corticosterone detected in adipose tissue, suggesting that the glucocorticoid effects led to attenuated NF-κB activation and unchanged TNFα. Similarly, our preliminary results in the adipose tissue of rats supplemented with liquid fructose for eight weeks suggest a lack of increase in inflammatory mediators, such as TNFα and monocyte chemotactic protein-1 (MCP-1). Interestingly, in adipose tissue samples of C57/BL6 mice fed a high-fat diet, the addition of 15% liquid fructose produced a more marked increase in these inflammatory markers (especially MCP-1) compared to animals fed only the high-fat diet [105].

### 6.4. Other Mechanisms

Sirtuin-1 (SIRT-1), a nicotinamide adenine dinucleotide (NAD+)-dependent deacetylase, has also been related to cancer development. SIRT-1 is a key molecular transducer involved in nutrient sensing in liver cells, modulating the function of many proteins that regulate cellular metabolism and survival. It also enhances hepatic fatty acid oxidation, protecting from diet-induced fatty liver, obesity and insulin-resistance [106]. With respect to its role in cellular survival, SIRT-1 deacetylates several molecules that regulate the cell cycle and apoptosis, such as p53, a tumor suppressor protein associated with the DNA damage repair system and the cell cycle arrest response. SIRT-1 inactivates p53 via deacetylation, which allows the proliferation of cells in the presence of damaged DNA, promoting tumor progression [107]. Accordingly, it has been shown that SIRT-1 upregulation promotes cell growth in human HCC tissues [108]. However, an opposite role for SIRT-1 as a tumor suppressor in HCC has also been proposed [109]. Thus, mice that received a single injection of the hepatocarcinogen, DEN, and were then exposed to a high-fat diet for 11 months had a 100% incidence of hepatic carcinomas, while mice overexpressing SIRT-1 were clearly protected [109]. The protective effect of SIRT-1 on HCC development may be related to the promotion of genome stability under stress conditions. A recent study showed higher expression and activity of SIRT-1 in human HCC tumors, compared with adjacent non-tumoral tissue [108]. We have previously reported that SIRT-1 expression and activity are reduced in the livers of 14-day fructose-supplemented rats [62]. We could speculate that the inhibition of SIRT-1 activity by fructose could represent another mechanism by which this carbohydrate may influence HCC development. Moreover, inhibition of SIRT-1 would induce m-TOR signaling [110], and mTOR activation may also lead to HCC through several mechanisms, as mentioned earlier.

Dietary fructose may also promote cancer growth through mechanisms related to the particular metabolic characteristics of cancerous cells. Since 1920, it has been known that cancer cells rely on anaerobic glycolysis rather than on mitochondrial glucose oxidation to obtain energy, a phenomenon termed the Warburg effect [111]. It has been suggested that fructose uncouples glycolysis from mitochondrial respiration, thus favoring a Warburg phenotype [35]. It has also been shown that fructose may facilitate tumorigenesis through induction of glucose-6-phosphate dehydrogenase, the key enzyme in the oxidative branch of the pentose phosphate pathway. Thus, in rats pre-treated with the carcinogen compound, N-nitrosomorpholine, for seven weeks and then fed with a diet supplemented with fructose in the drinking water (12% w/v), there was an increase in the incidence of HCC that correlated with increased expression of G6PDH [85]. The authors proposed that the increase in this enzyme would support cell growth and proliferation through the generation of ribose 5-phosphate and NADPH.

Other mechanisms by which fructose can promote cancer progression relate to the ability of this carbohydrate to increase the production of reactive oxygen species (ROS) or to induce direct DNA damage. ROS generation can contribute to hepatic carcinogenesis through the promotion of liver fibrosis [112] or direct damage of cellular lipids, proteins or DNA [113]. *In vitro* studies in hepatocytes showed that fructose is cytotoxic at high concentrations, through oxidative stress via the generation of ROS and H_2_O_2_, and that fructose cytotoxicity was prevented by ROS scavengers [114]. In addition, *in vivo* studies have shown that fructose can induce ROS formation in the liver: mice fed a diet high in fat and high in fructose had significantly higher hepatic ROS levels compared to both high fat- and chow-fed mice [115]. In this study, increased hepatic ROS was associated with collagen deposition and fibrosis in this organ. In another recent study, male rats were 65% fructose in solid form for eight weeks showed an increase in hepatic thiobarbituric acid-related substances (TBARS) and conjugated dienes, indicative of lipid peroxidation and oxidative stress, together with a significant decrease in antioxidant defenses, including superoxide dismutase (SOD) and vitamin C [116]. Finally, direct DNA damage by fructose has only been observed in *in vitro* studies and at high concentrations [117,118].

**Figure 1:**
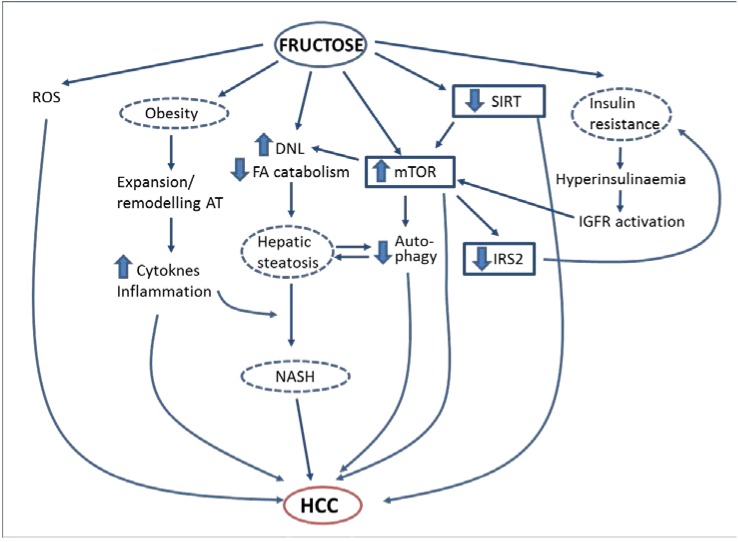
Proposed mechanisms by which fructose may promote hepatocellular carcinoma.

AT, adipose tissue; DNL, *de novo* lipogenesis; FA, fatty acids; HCC, hepatocellular carcinoma; IGFR, insulin growth factor receptor superfamily; IRS2, insulin receptor substrate 2; mTOR, mammalian target of rapamycin; NASH, non-alcoholic steatohepatitis; ROS, reactive oxygen species; SIRT, sirtuin-1.

## 7. Conclusions

In summary, metabolic disorders caused by excessive simple sugar, and specifically fructose, consumption (insulin resistance, obesity, NAFLD) may lead to the development of HCC (Figure 1). It is thus logical to propose that a diet containing an excessive amount of fructose is one of the environmental risk factors for HCC. However, the epidemiological evidence confirming this link is still scarce, so more studies are warranted to confirm this hypothesis. In addition, studies in animal models chronically supplemented with fructose (or with sucrose or high-fructose corn syrup) are needed to firmly establish the role of these dietary components in HCC development and to clearly unravel the molecular mechanisms involved. If the relationship is confirmed, one can envisage a simple strategy to reduce the burden of HCC, involving reducing the amount of added sugars in the diet. Moreover, a deep knowledge of the pathways linking sugar consumption with HCC development may lead to the discovery of new molecular targets for the treatment of this type of cancer.
